# Positive regulation of innate immune response by miRNA-let-7a-5p

**DOI:** 10.3389/fgene.2022.1025539

**Published:** 2023-01-06

**Authors:** Mayumi Ueta, Hiromi Nishigaki, Seitaro Komai, Katsura Mizushima, Risa Tamagawa-Mineoka, Yuji Naito, Norito Katoh, Chie Sotozono, Shigeru Kinoshita

**Affiliations:** ^1^ Department of Ophthalmology, Kyoto, Japan; ^2^ Department of Human Immunology and Nutrition Science, Kyoto, Japan; ^3^ Department of Dermatology, Kyoto, Japan; ^4^ Department of Frontier Medical Science and Technology for Ophthalmology, Kyoto Prefectural University of Medicine, Kyoto, Japan

**Keywords:** atopic keratoconjunctivitis, atopic dermatitis, let-7a-5p, plasma, TLR3

## Abstract

**Background:** We have hypothesized that different factors are involved in the severity of ACD and AD because some but not all patients with atopic dermatitis (AD) present with allergic conjunctival disease (ACD) including severe types such as atopic keratoconjunctivitis (AKC) with/without giant papillae. We previously reported that plasma miR-628-3p was up-regulated in AD with severe ACD, but not in severe AD without severe ACD or in our healthy controls. In this study, to investigate the pathogenesis of AD with and without severe ACD, we performed comprehensive plasma miRNA analysis and studied the function of some miRNAs which were significantly up-regulated in ACD.

**Methods:** Transcriptomics analysis of miRNA was performed using the microarray platform from the plasma of nine individuals (AD, severe ACD, controls: *n* = 3 each). To confirm up-regulation of the 12 miRNAs of the eight miRNA groups we focused on, we performed quantitative miRNA polymerase chain reaction (PCR) assays using 80 plasma samples (AD: 23, severe ACD: 17, controls: 40). To study the function of the eight miRNAs which were significantly up-regulated in ACD, we transfected their mimic to THP-1 cells, a monocyte cell line, and performed comprehensive gene expression analysis of them. The up-regulation of gene expression of interest in transfected THP-1 cells with the hsa-let-7a-5p miRNA mimic was confirmed by quantitative RT-qPCR assay.

**Results:** Quantitative miRNA PCR assays showed that hsa-let-7a-5p, hsa-let-7days-3p, hsa-let-7e-5p, and hsa-miR-151a-5p were significantly up-regulated in both AD-ACD^**+**^ and AD-ACD^**-**^ as were hsa-miR-130a-3p, hsa-miR-151a-3p, has-miR-27b-3p, and hsa-miR-146a-5p in AD-ACD^**+**^ but not in AD-ACD^**-**^. The functions of each miRNA were investigated by comprehensive gene expression analysis of THP-1 cells transfected with each miRNA mimic. Of the eight miRNAs, hsa-let-7a-5p, hsa-let-7e-5p, has-miR-27b-3p, and hsa-miR-146a-5p mimic-transfected THP-1 cells showed the up-regulation of CXCL10 (IP-10; interferon gamma-induced protein 10), which might be one of the innate immune-related genes. Quantitative RT-qPCR assays of transfected THP-1 cells with the hsa-let-7a-5p miRNA mimic showed that the 17 genes up-regulated more than 10-fold in the comprehensive gene expression analysis, and TLR3, RIG-I, and MDA5, important innate immune-related genes, were significantly up-regulated. TNFSF13B, AIM2, USP41, STAP1, GBP4, CCL8, and IFI27, reportedly down-regulated by the hsa-miR-628-3p mimic, were also significantly up-regulated in the transfected cells.

**Conclusion:** Hsa-let-7a-5p, which was significantly up-regulated in AD-ACD^**+**^ and AD-ACD^**-**^, could positively regulate the important innate immune-related genes such as TLR3, RIG-I, and MDA5. It is possible that in an allergic disease such as atopic keratoconjunctivitis and/or dermatitis, innate immune responses might be positively regulated by hsa-let-7a-5p in the plasma.

## Introduction

MicroRNAs (miRNAs), which, in their mature form, are small non-coding RNAs consisting of an average of about 22 nucleotides, function as endogenous regulators of the expression of many genes. miRNAs usually bind to complementary sequences in untranslated regions of target mRNAs and inhibit their expression. They play a role in cell/tissue functions including the immune response, apoptosis, and cell differentiation and are involved in the pathogenesis of various human diseases (Ardekani and Naeini, 2010).

Elsewhere (Ueta et al., 2021a), we reported that miR-628-3p was up-regulated in the plasma of patients with Stevens–Johnson syndrome (SJS)/toxic epidermal necrolysis (TEN) with severe ocular complications (SOCs) in the chronic stage and that miR-628-3p negatively regulated innate immunity by suppressing pathogen-associated molecular patterns (PAMPs) such as TLR3, RIG-I, and MDA5.

We also reported that TLR3 regulated allergic reactions such as allergic conjunctivitis (Ueta et al., 2009) and contact and atopic dermatitis (Nakamura et al., 2015; Yasuike et al., 2017), because these allergic reactions were reduced in TLR3 knock-out mice.

In our clinic, we encountered patients with severe ocular surface inflammatory diseases, e.g., SJS/TEN with SOC, severe allergic conjunctival disease (ACD) such as atopic and/or vernal keratoconjunctivitis with shield ulcers.

Another study (Ueta et al., 2021b) showed that plasma miR-628-3p was up-regulated in atopic dermatitis (AD) with ACD (AD-ACD^+^) with giant papillae but not in severe AD-ACD^-^ or in healthy controls. This suggests that the plasma miR-628-3p level represents a marker to predict severe ACD in AD patients.

To investigate the pathogenesis of AD-ACD^+^ and AD-ACD^-^, we performed comprehensive miRNA analysis of plasma from patients with AD-ACD^+^ and AD-ACD^-^ and healthy controls using microarrays. We also confirmed the up-regulation of miRNAs of interest by real-time quantitative polymerase chain reaction (RT-qPCR) assays. Moreover, to study the function of some miRNAs which was significantly up-regulated in ACD, we transfected its mimic to THP-1 cells, a monocyte cell line, respectively, and performed comprehensive gene expression analysis. The up-regulation of gene expression of interest in transfected THP-1 cells with the hsa-let-7a-5p miRNA mimic was confirmed by quantitative RT-qPCR assay.

## Materials and methods

### Human plasma

This study was approved by the Institutional Review Board of Kyoto Prefectural University of Medicine. All experimental procedures were conducted in accordance with the tenets of the Declaration of Helsinki. Written informed consent was obtained from all patients after they were given a detailed explanation of the purpose of the research and the experimental protocols.

### Microarray profiling of miRNAs

As previously reported and as recommended by the manufacturers, the total RNA was isolated and purified from plasma using the miRNeasy serum/plasma kit (Qiagen, Tokyo, Japan). Affymetrix GeneChip miRNA 4.0 arrays were used for miRNA profiling. Total RNA was labeled with the FlashTag™ Biotin HSR RNA labeling kit (Affymetrix, Inc., USA) (Ueta et al., 2020; Ueta et al., 2021a). The samples were hybridized on GeneChip miRNA 4.0 arrays (Affymetrix) for 18 h at 48°C. The arrays were washed to remove non-specifically bound nucleic acids and stained on Fluidics Station 450 (Affymetrix)) and then scanned on a GeneChip Scanner 3000 7G system (Affymetrix).

### Quantitative miRNA PCR

As previously reported (Ueta et al., 2020; Ueta et al., 2021a), we used TaqMan MicroRNA reverse transcription kits (Applied Biosystems, Vilnius, Lithuania**)** for the RT reaction. Quantitative miRNA PCR assays were performed on a StepOnePlus instrument (Applied Biosystems) according to the manufacturer’s instructions. The primers and probes were purchased from Applied Biosystems.

The universal master mix and the specific primer and probe mix included in predesigned TaqMan MicroRNA assays were hsa-let-7a-5p, ID: 000,377; hsa-miR-150-5p, ID: 002,637; hsa-miR-451b, ID: 464,419_mat; hsa-miR-151a-3p, ID: 002,254; hsa-miR-151a-5p, ID: 002,642; hsa-miR-199a-3p, ID: 002,304; hsa-miR-30a-5p, ID: 000,417; hsa-miR-126-3p, ID: 002,228; hsa-miR-130a-3p, ID: 000,454; hsa-miR-146a-5p, ID: 000,468; hsa-miR-30days-5p, ID: 000,420; hsa-miR-744-5p, ID: 002,324; hsa-miR-106a-5p, ID: 002,169; hsa-miR-17-5p, ID: 002,308; hsa-miR-130b-3p, ID: 000,456; and hsa-let-7days-3p, ID: 001,178 (Applied Biosystems). microRNA expression was calculated with the method described in the previous paragraphs. Spike-in cel-miR-39 (miR-39, ID: 000,200/, P/N:P/N:4,440,887, Applied Biosystems) was used for normalization.

### Transfections with the miRNA mimics significantly up-regulated in ACD

THP-1 cells were purchased from the JCRB cell bank (Osaka, Japan). For transfection with the miRNA mimic and for RT-qPCR, we cultured THP-1 cells as recommended by the manufacturer; 2-day stimulation was with 100 ng/ml PMA (Sigma-Aldrich, Saint Louis, MO). The tansfected THP-1 cells were used in subsequent procedures as previously reported (Ueta et al., 2021a). The eight miRNA mimics and the controls for miRNA-let-7a-5p, let-7days-3p, let-7e-5p, 146a-5p, 130a-3p, 151a-3p, and 27b-3p were from Applied Biosystems. The mimic and the negative control were mixed with lipofectamine RNAiMAX (Invitrogen, Carlsbad, CA) and added for 24 h to the THP-1 cells at 80% confluence.

### Comprehensive gene expression analysis of THP-1 cells with the eight up-regulated miRNAs, respectively

Gene expression profiles were investigated using a high-density oligonucleotide probe array [GeneChip^®^, Human Clariom S Array (Affymetrix)] as in our previous report (Ueta et al., 2020; Ueta et al., 2021a). Total RNA was extracted with the QIAGEN RNeasy kit (Qiagen, Valencia, CA). We used approximately 337,100 probe sets covering more than 20,800 genes. We followed Affymetrix instructions throughout. Scanned microarray images were obtained on a 3000 7G GeneChip Scanner (Affymetrix) using the default settings. Images were visually inspected to detect hybridization artifacts.

### Quantitative RT-PCR

Total RNA was isolated using the RNeasy mini kit according to the manufacturer’s instructions. For the RT reaction we used ReverTraAce (TOYOBO, Japan). RT-qPCR assays were performed on a StepOnePlus instrument (Applied Biosystems) according to the manufacturer’s instructions. The primers and probes were purchased from Applied Biosystems (RSAD2: Hs00369813, CXCL10: Hs00171042, IFI44L: Hs00915292, TRIM22: Hs01001179, CXCL11: Hs00171138, IFIT2: Hs01922738, IFIT1: Hs0 3,027,069, MX1: Hs00895608, MX2: Hs01550811, OAS2: Hs00942643, HERC5: Hs00180943, DDX58: Hs00204833, TNFSF10: Hs00921974, IFITM1: Hs00705137, TLR3: Hs00152933, and IFIH1: Hs00223420). Quantification data were normalized to the expression of the housekeeping gene GAPDH.

### Data analysis

For microarray analysis, we used the ANOVA *p* value to record significant differences between the patients and controls. Data from quantitative miRNA PCR- and RT-qPCR assays were expressed as the mean ± SE. Quantitative miRNA PCR and RT-qPCR assays were evaluated by Student’s *t*-test using Microsoft Excel. Quantitative miRNA PCR assays were also evaluated by the Tukey–Kramer method for multiple comparison test.

## Results

### Comprehensive plasma miRNA analysis and quantitative miRNA PCR analysis

For microarrays, we used nine plasma samples (AD-ACD^+^ with giant papillae, severe AD-ACD^-^, and healthy controls, *n* = 3 each).

Comparison with the controls revealed a more than 5-fold up-regulation in 183 miRNAs from patients with AD-ACD^+^ (*p* < 0.05 by ANOVA). Also, 233 miRNAs were significantly up-regulated (>5x) in AD-ACD^-^ compared with the controls (*p* < 0.05 by ANOVA). The GEO repository shows our data of comprehensive miRNA analysis of plasma: GSE217232 (https://www.ncbi.nlm.nih.gov/geo/query/acc.cgi?acc=GSE217232).

**FIGURE 1:**
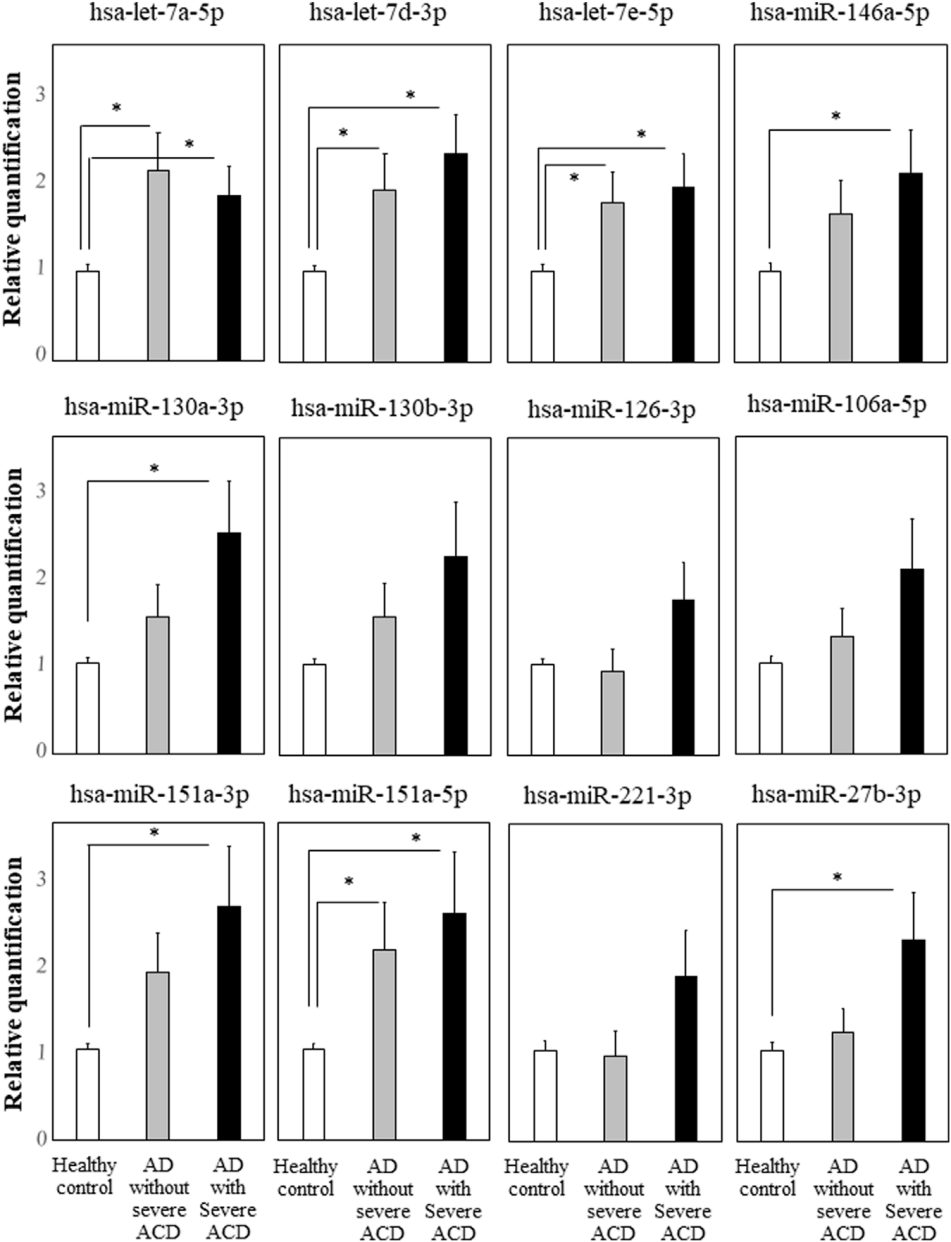
Quantitative miRNA PCR analysis in plasma quantification data was normalized to the expression of the internal control (miR-39). The *Y*-axis shows the increase in specific miRNA over the control samples. Data are the mean ± SEM (healthy controls *n* = 40, AD without severe ACD *n* = 17, and AD with severe ACD *n* = 23). *p < 0.05.

We focused on the 12 miRNAs in the eight miRNA groups listed in Table 1. To confirm their up-regulation, we performed quantitative miRNA PCR assay using plasma samples from AD-ACD^+^ (*n* = 23), AD-ACD (*n* = 17) patients, and healthy controls (*n* = 40). As shown in Figure 1, compared with the control, among 12 miRNAs, four were significantly up-regulated in both AD-ACD^+^ and AD-ACD^-^ patients, while four were significantly up-regulated only in AD-ACD^+^. In the multiple comparison test, only miRNA let-7a-5p was significantly up-regulated in both AD-ACD^+^ and AD-ACD^-^ patients compared with controls.

**TABLE 1:** Result of the comprehensive miRNA analysis of plasma.

miRNA group which consists miRNAs more than 15	Number of miRNAs which show more than 5-fold in comparison		Human miRNA
	AD-ACD+ vs. controls	AD-ACD- vs. controls	
let-7	41	60	hsa-let-7a-5p, hsa-let-7days-3p, hsa-let-7e-5p
miR-130	30	1	hsa-miR-130a-3p, hsa-miR-130b-3p
miR-151	17	16	hsa-miR-151a-3p, hsa-miR-151a-5p
miR-146	15	1	hsa-miR-146a-5p
miR-126	15	0	hsa-miR-126-3p
miR-106	15	17	hsa-miR-106a-5p
miR-221	0	28	hsa-miR-221-3p
miR-27	0	20	hsa-miR-27b-3p

### Comprehensive gene expression analysis of THP-1 cells transfected with each candidate hsa-miRNA mimic

We focused the eight *miRNAs,* miRNA-let-7a-5p, let-7days-3p, let-7e-5p, 146a-5p, 130a-3p, 151a-3p, 151a-5p, and 27b-3p, that significantly up-regulated in AD-ACD^+^ patients compared with controls. To investigate their functions, we performed comprehensive gene expression analysis of THP-1 cells transfected with each miRNA mimic, respectively. The quantitative miRNA PCR assay could confirm that in transfected cells with each miRNA mimic of miRNA-let-7a-5p, let-7d-3p, let-7e-5p, 146a-5p, 130a-3p, 151a-3p, 151a-5p, and 27b-3p, each miRNA was significantly up-regulated, respectively (Supplementary Figure S1 shows the up-regulation of hsa-let-7a-5p by its mimic transfection as representative data).

Supplementary Tables S1, S2 list the genes whose expression was up-regulated more than 3-fold in THP-1 cells transfected with each hsa-miRNA mimic compared with controls.

The 84 genes were up-regulated more than 3-fold in the THP-1 cells transfected with miRNA-let-7a-5p mimic (Supplementary Table S1)*,* while the 10 genes in that were transfected with miR-151a-3p mimic, the nine genes in that were transfected with miRNA-let-7e-5p mimic, the seven genes in that were transfected with miRNA-let-7d-3p mimic, miR-146a-5p, and miR-27b-3p, respectively, the six genes in that were transfected with miR-151-5p mimic, and the two genes in that were transfected with miR-130a-3p mimic (Supplementary Table S2). Of the eight miRNA, hsa-let-7a-5p, hsa-let-7e-5p, has-miR-27b-3p, and hsa-miR-146a-5p mimic-transfected THP-1 cells showed the up-regulation of CXCL10 (IP-10; interferon gamma-induced protein 10), which might be one of innate immune-related genes. Moreover, the transfection with miRNA-let-7a-5p mimic to THP-1 cells strongly affected their gene expression.

GEO repository shows our data of comprehensive gene expression analysis as follow; THP-1 cells transfected with the hsa-let-7a-5p miRNA mimic: GSE217224 (https://www.ncbi.nlm.nih.gov/geo/query/acc.cgi?acc=GSE217224). THP-1 cells transfected with the hsa-miR-27b-3p and miR-151a-5p miRNA mimic: GSE217969 (https://www.ncbi.nlm.nih.gov/geo/query/acc.cgi?acc=GSE217969). THP-1 cells transfected with the hsa-let-7d-3p, let-7e-5p, miR-146a-5p, miR-130a-3p, and miR-151a-3p miRNA mimic: GSE217971 (https://www.ncbi.nlm.nih.gov/geo/query/acc.cgi?acc=GSE217971).

### RT-qPCR analysis of THP-1 cells transfected with the hsa-let-7a-5p miRNA mimic

We focused on the THP-1 cells transfected with the hsa-let-7a-5p miRNA mimic and performed RT-qPCR analysis to confirm the up-regulation of gene expression showed in the comprehensive gene expression analysis.

Elsewhere (Ueta et al., 2021b), we reported that plasma miR-628-3p was significantly up-regulated in AD-ACD^+^ with giant papillae, suggesting that its plasma level represents a marker to predict severe ACD in AD patients and that the hsa-miR-628-3p mimic down-regulated PAMPs such as TLR3, RIG-I, and MDA5 and 19 genes (STAP1, IFI44L, CXCL11, TNFSF10, AIM2, RSAD2, IFITM1, CXCL10, CCL8, TRIM22, HERC5, IFI27, IFIT2, GBP4, IFIT1, IDO1, HESX1, TNFSF13B, and USP41).

The 84 up-regulated genes (Supplementary Table S1) included DDX58 (also known as RIG-I), IFIH1 (also known as MDA5), and 17 of the 19 genes listed in the previous paragraphs but not IDO1 and HESX1. RIG-I, MDA5, and TLR3 are receptors of double-stranded RNA, they also induce interferon-related genes.

**FIGURE 2:**
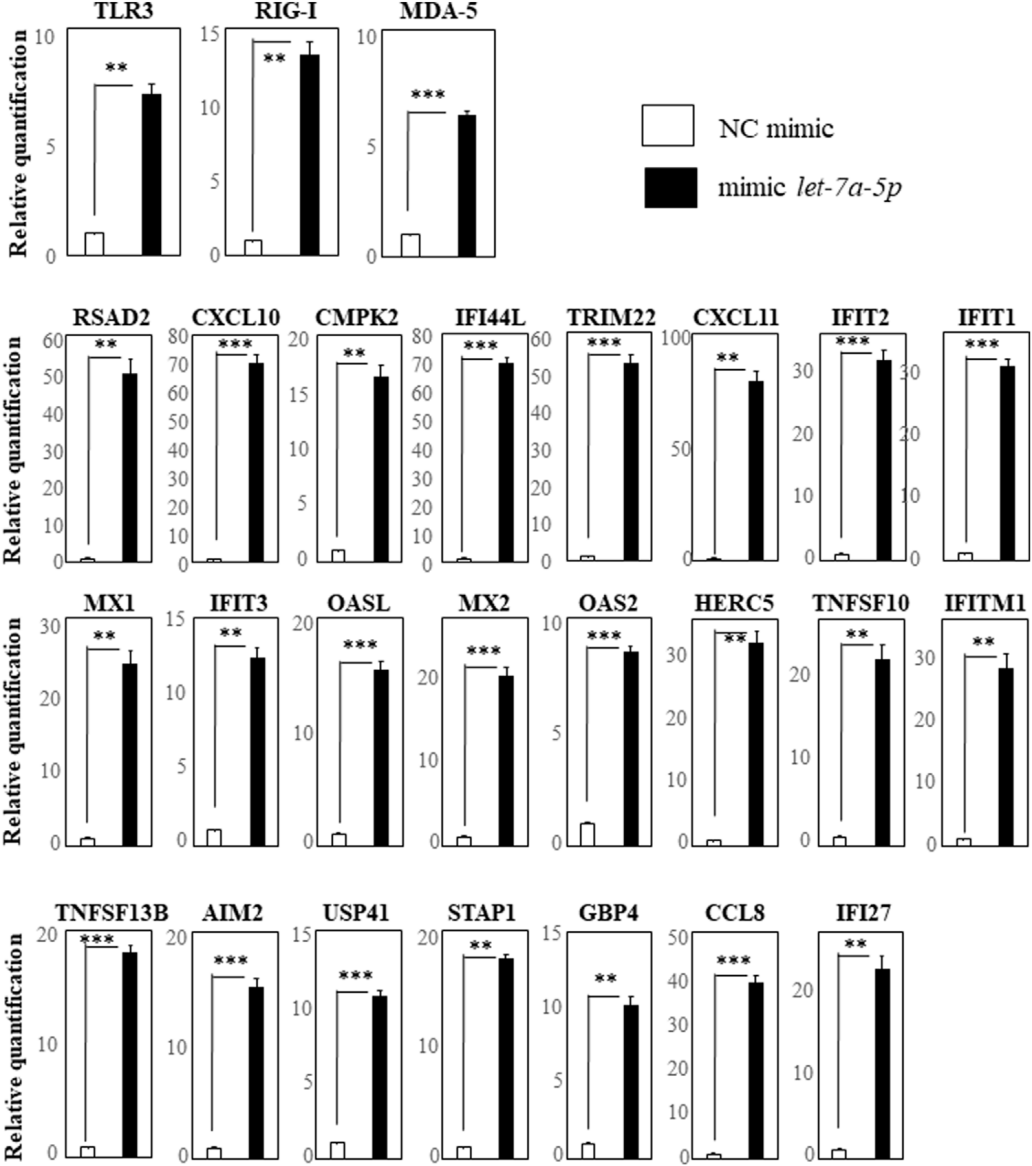
RT-qPCR analysis of genes in THP-1 cells transfected with hsa-let-7a-5p miRNA quantification data were normalized to the expression of the house-keeping gene GAPDH. The *Y*-axis shows the increase in specific mRNA over the control samples. Representative data of three experiments are given. Data are the mean ± SEM (each group *n* = 4). Mimic NC: mimic control; mimic let-7a-5p: mimic of hsa-let-7a-5p miRNA.**p < 0.005; ***p < 0.0005.

We first selected the 17 genes (RSAD2, CXCL10, CMPK2, IFI44L, TRIM22, CXCL11, IFIT2, IFIT1, MX1, IFIT3, OASL, MX2, OAS2, HERC5, DDX58 (RIG-I), TNFSF10, and IFITM1) with more than 10-fold up-regulation by hsa-let-7a-5p miRNA-mimic transfection revealed by comprehensive gene expression analysis. We also selected TLR3 and MDA5, receptors of double-stranded RNA as same as RIG-I, and TNFSF13B, AIM2, USP41, STAP1, GBP4, CCL8, and IFI27 that had been reported to be down-regulated by the hsa-miR-628-3p mimic (Ueta et al., 2021b). We investigated their up-regulation by performing RT-qPCR assays and found that, compared with the negative control, the expression of TLR3, RIG-I, and MDA5 was significantly up-regulated in cells transfected with the hsa-let-7a-5p mimic (Figure 2, top row). In addition, 16 genes that, based on our comprehensive gene expression analysis, were up-regulated more than 10-fold by hsa-let-7a-5p miRNA-mimic transfection were significantly up-regulated in cells transfected with the let-7a-5p mimic (Figure 2, center rows). The seven genes that have been reported to be down-regulated by the hsa-miR-628-3p mimic (Ueta et al., 2021b) were significantly up-regulated in cells transfected with the let-7a-5p mimic (Figure 2, bottom row).

## Discussion

We subjected plasma samples from patients with AD-ACD^+^ and AD-ACD^-^ and from healthy controls to comprehensive miRNA analysis.

We focused on the eight *miRNAs*, miRNA-let-7a-5p, let-7d-3p, let-7e-5p, 146a-5p, 130a-3p, 151a-3p, 151a-5p, and 27b-3p, significantly up-regulated in AD-ACD^+^ patients compared with controls in quantitative miRNA PCR analysis, and performed comprehensive gene expression analysis of THP-1 cells transfected with each miRNA mimic, respectively. Interestingly, of the eight miRNAs, hsa-let-7a-5p, hsa-let-7e-5p, has-miR-27b-3p, and hsa-miR-146a-5p mimic-transfected THP-1 cells showed a more than three-fold up-regulation of CXCL10. CXCL10 is also called as IP-10 (interferon gamma-induced protein 10) and one of the important innate immune-related genes.

We also analyzed the network of these eight miRNAs though gene expressions using miRWalk website (http://mirwalk.umm.uni-heidelberg.de/). Supplementary Figure S2 shows the network of them and might suggest that four miRNAs (hsa-let-7a-5p, hsa-let-7e-5p, has-miR-27b-3p, and hsa-miR-146a-5p), which were significantly up-regulated in both patients of AD-ACD^+^ and AD-ACD^-^, might make strong relation.

hsa-let-7a-5p was significantly up-regulated in both patients of AD-ACD^+^ and AD-ACD^-^ and its mimic-transfected THP-1 cells strongly affected their gene expression; comprehensive gene expression analysis of THP-1 cells transfected with the hsa-let-7a-5p mimic showed that 17 genes were up-regulated more than 10-fold. RT-qPCR assays revealed their significant up-regulation and that of TLR3, RIG-I, and MDA5, important innate immune-related genes. TNFSF13B, AIM2, USP41, STAP1, GBP4, CCL8, and IFI27, reported to be down-regulated by the hsa-miR-628-3p mimic (Ueta et al., 2021b), were also significantly up-regulated in the transfected cells.

Liu et al. (2016) and Liu et al. (2018) identified let-7a-5p as a potential tumor suppressor in many types of cancer. In acute myeloid leukemia, let-7a-5p was up-regulated (Fasihi-Ramandi et al., 2018). According to Matsuura et al. (2018), the plasma level of let-7a-5p was inversely correlated with the severity of hepatic fibrosis in patients with chronic hepatitis C. Liu and Jiang (2020) reported that it suppressed the epithelial–mesenchymal transition in TGF-β-induced human lens epithelial cells.

Weidner et al. gave an overview of miRNAs in allergic diseases, including AD, allergic rhinitis, and asthma (Weidner et al., 2021), and described the up-regulation of miR-21, miR-146a, miR-155, miR-151a, miR-143, miR-124, miR-223, and miR-10a in AD patient samples. Of these miRNAs, we also detected miR-146a and miR-151a; miR-146a-5p and miR-151a-3p were significantly up-regulated in AD with ACD but not in AD without ACD, and miR-151a-5p was significantly up-regulated both in AD with and without ACD. We focused on severe ACD and investigated miRNA in plasma of AD. This might be a first report which divided AD to two groups focused on with or without severe ACD and compared their miRNA. miR-146a was reported to alleviate chronic skin inflammation in atopic dermatitis through suppression of innate immune responses in keratinocytes (Rebane et al., 2014). It might be certain that miR-146a could regulate innate immune responses, because, in our study, using THP-1 cells, miR-146a-5p mimic up-regulated innate immune-related genes such as CXCL10, CXCL11, CCL2 and CCL8. MiR-150a was reported to be involved in the pathogenesis of atopic dermatitis by regulating interleukin-12 receptor beta2 (Chen et al., 2018); however, in our study, using THP-1 cells, miR-151a-3p or miR-151a-5p mimic did not regulate interleukin-12 related genes or innate immune-related genes.

There are few reports on the association between let-7a-5p in the plasma and allergic diseases. We think that ours is the first report showing that let-7a-5p regulates the innate immune response *via* the up-regulation of PAMPs such as TLR3, MDA5, and RIG-I. TLR3 was involved in the pathogenesis of AD (Nakamura et al., 2015; Yasuike et al., 2017) and ACD (Ueta et al., 2009), so the up-regulation of let-7a-5p may play a role in the development of allergic diseases such as AD and ACD.

We noted that hsa-let-7a-5p positively regulated the innate immune-related genes and that its function was opposite to the negative regulation exerted by hsa-miR-628-3p. In AD-ACD^+^ and AD-ACD^-^, hsa-let-7a-5p, a positive regulator of the innate immune response, was significantly up-regulated, while hsa-miR-628-3p, a negative regulator, was significantly up-regulated only in AD-ACD^+^, suggesting that the balance between hsa-let-7a-5p and hsa-miR-628-3p might play a role in the elicitation of severe ACD.

However, this study might have a limitation due to the small number of cases and controls. In the future, further studies with expanded cases and controls are needed.

Allergic diseases include AD, ACD, asthma, and allergic rhinitis. We suggested (Ueta et al., 2021b) that different factors may be involved in the severity of different allergic diseases because their combination varies; some patients present with AD and ACD, some with only asthma, and some with asthma and allergic rhinitis.

We think that, to identify factors involved in the severity of each allergic disease, more studies are needed for their early diagnosis and prediction and for the development of novel therapies.

## Data Availability

The datasets presented in this study can be found in online repositories. The names of the repository/repositories and accession number(s) can be found in the article/Supplementary Material.

## Abbreviations

AIM2, absent in melanoma 2; CCL8, chemokine (C-C motif) ligand 8; CMPK2, cytidine monophosphate (UMP-CMP) kinase 2, mitochondrial; CXCL10, chemokine (C-X-C motif) ligand 10; CXCL11, chemokine (C-X-C motif) ligand 11; DDX58 (RIG-I, DEAD (Asp-Glu-Ala-Asp) box polypeptide 58; GBP4, guanylate binding protein 4; HERC5, HECT, and RLD domain-containing E3 ubiquitin protein ligase 5; IFI27, interferon, alpha-inducible protein 27; IFI44L, interferon-induced protein 44-like; IFIH1 (MDA5), interferon induced with helicase C domain 1; IFIT1, interferon-induced protein with tetratricopeptide repeats 1; IFIT2, interferon-induced protein with tetratricopeptide repeats 2; IFIT3, interferon-induced protein with tetratricopeptide repeats 3; IFITM1, interferon-induced transmembrane protein 1; MX1, MX dynamin-like GTPase 1; MX2, MX dynamin-like GTPase 2; OAS2, 2′-5′-oligoadenylate synthetase 2; OASL, 2-5-oligoadenylate synthetase-like; RSAD2, radical S-adenosyl methionine domain-containing 2; STAP1, signal transducing adapter family member 1; TLR3, toll-like receptor 3; TNFSF10, tumor necrosis factor (ligand) superfamily, member 10; TNFSF13B, tumor necrosis factor (ligand) superfamily, member 13b; TRIM22, tripartite motif-containing 22; USP41, ubiquitin specific peptidase 41.
