# Supplementary figures and images for: Positive regulation of innate immune response by miRNA-let-7a-5p
Source: Front Genet. 2023 Jan 6;13:1025539. doi: 10.3389/fgene.2022.1025539 (PMC9858567; doi:10.3389/fgene.2022.1025539)

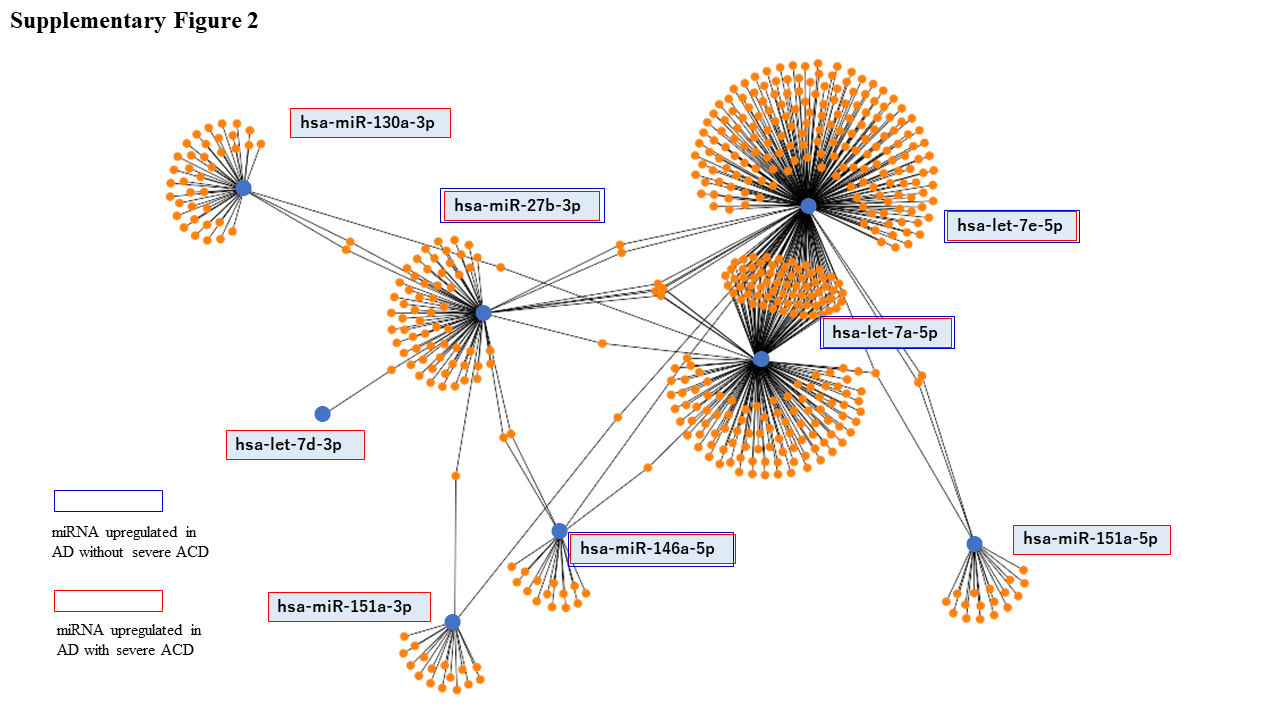

Supplement: Supplementary file 2 [file Image2.TIF]

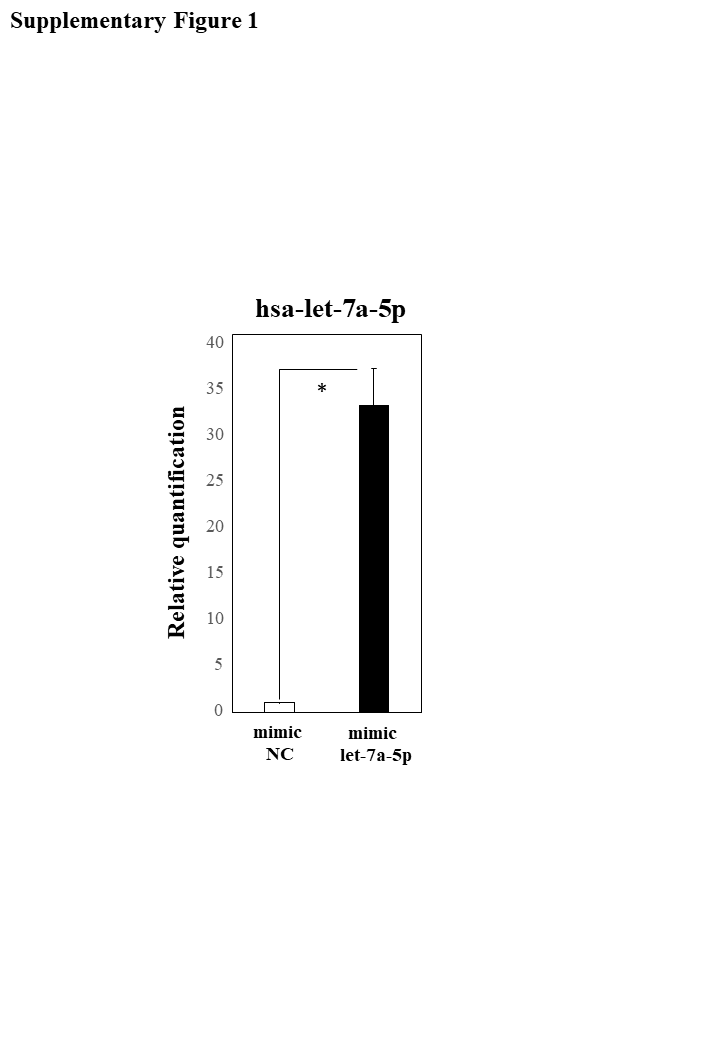

Supplement: Supplementary file 3 [file Image1.TIF]
