# Supplementary material for: Positive regulation of innate immune response by miRNA-let-7a-5p
Source: Front Genet. 2023 Jan 6;13:1025539. doi: 10.3389/fgene.2022.1025539 (PMC9858567; doi:10.3389/fgene.2022.1025539)
Supplement: Supplementary file 4 [file Table3.DOCX]

**Positive regulation of innate immune response by miRNA let-7a-5p**

Mayumi Ueta^1^, Hiromi Nishigaki^1^, Seitaro Komai^1^, Katsura Mizushima^2^, Risa Tamagawa-Mineoka^3^, Yuji Naito^2^, Norito Katoh^3^, Chie Sotozono^1^, Shigeru Kinoshita^4^

^1^Department of Ophthalmology, ^2^Department of Molecular Gastroenterology and Hepatology, ^3^ Department of Dermatology, ^4^Department of Frontier Medical Science and Technology for Ophthalmology, Kyoto Prefectural University of Medicine, Kyoto, Japan

**CORRESPONDING AUTHOR**

Mayumi Ueta, M.D., Ph.D., Associate Professor

Department of Ophthalmology, Kyoto Prefectural University of Medicine

465 Kajiicho, Hirokoji, Kawaramachi, Kamigyoku, Kyoto, 602-0841, Japan

Phone: 81-75-251-5578, Fax: 81-75-251-5663, e-mail: mueta@koto.kpu-m.ac.jp

**Supplementary Figure Legends**

***Supplementary Figure 1.***

***Quantitative miRNA PCR analysis of hsa-let-7a-5p in THP-1 cells transfected with the hsa-let-7a-5p mimic***

Quantification data were normalized to the expression of the internal control (miR-39). The Y axis shows the increase in specific miRNA over the control samples. Representative data of 4 experiments. Data are the mean ± SEM (each group n=4). mimic NC: mimic control, mimic let-7a-5p: mimic of hsa-let-7a-5p.

*p < 0.05

***Supplementary Figure 2.***

The network of 8 *miRNAs,* miRNA-let-7a-5p, let-7d-3p, let-7e-5p, 146a-5p, 130a-3p, 151a-3p, 151a-5p, 27b-3p using miRWalk website.
